# Emotional processing as mechanism of change in brief good psychiatric management for borderline personality disorder: results of a randomized controlled trial

**DOI:** 10.1186/s12888-024-06370-2

**Published:** 2024-12-18

**Authors:** Ueli Kramer, Loris Grandjean, José Blanco Machinea, Hélène Beuchat, Setareh Ranjbar, Yves de Roten, Jean-Nicolas Despland, Philippe Conus, Stéphane Kolly

**Affiliations:** 1 University Institute of Psychotherapy, Department of Psychiatry, University of Lausanne and Lausanne University Hospital, Route de Cery 1, CH-1008 Prilly-Lausanne, Switzerland; 2 General Psychiatry Service, Department of Psychiatry, Lausanne University Clinic and University of Lausanne, Lausanne, Switzerland; 3 Department of Psychiatry, Lausanne University Clinic and University of Lausanne, Lausanne, Switzerland; 4 Department of Psychology, University of Windsor, Windsor, Canada

**Keywords:** Brief good psychiatric management, Psychiatric treatment, Randomized controlled trial, Mechanisms of change, Borderline personality disorder, Emotional processing

## Abstract

**Background:**

Borderline Personality Disorder (BPD) is one of the most frequent, severe, mental conditions and is associated with a serious burden of disease. Treatment for patients with BPD involves structured psychotherapy. In addition and in order to improve access to care, psychiatric treatments are available. So far, it remains unclear if brief psychiatric intervention according to Good Psychiatric Management (GPM) produces a reliable effect on a variety of clinical outcomes. The assessment of mechanisms of change contributes to focus the treatment on the essential ingredients of change. The current study aims to demonstrate the emotional processing as a mechanism of change, assessed in an ecologically valid experimental context, of brief GPM.

**Methods:**

The present two-arm randomized controlled study aims at testing the effects (i.e., reduction in borderline symptoms) and emotional processing as mechanism of change of a brief psychiatric treatment (10 sessions over 4 months), compared with treatment as usual. Participants are *N* = 76 patients with BPD who are either randomized to a 4 month GPM or a 4 month TAU without any BPD-specific content. All patients undergo assessments of borderline symptoms using ZAN-BPD and experiential enactment for assessment of emotional processing, at intake, 2 months and 4 months (discharge).

**Results:**

The effect of a brief version of GPM is not different from the effect of TAU on the total score of the ZAN-BPD at 4 month (*d* = 0.04). At the level of the secondary effects, GPM is more effective in reducing relationship problems on the ZAN-BPD sub-scale (*F*(1, 61) = 5.53; *p* = .022, *d* = 0.52), and in reducing impulsivity and social problems, and it increases treatment retention. Change in emotional processing between intake and month 2 mediates the reduction on the ZAN-BPD subscale relationship problems assessed between months 2 and 4, in an ecologically valid experimental context.

**Conclusions:**

Overall, brief GPM is not different from TAU in terms of reduction in borderline symptoms, but it may produce reduction in relationship problems between months 2 and 4 into the brief intervention. Change in emotional processing in an experimental context may function as mechanism of change of brief GPM. This study is in line with the top priority of establishing efficacy of brief interventions for BPD and proposes an evidence-based explanation for efficacy. These results should help disseminate brief psychiatric treatments for BPD, which contribute to reduce the societal burden related with BPD.

**Trial registration:**

Clinical Trials NCT03717818 (date of registration of Abstract October 24th, 2018). Protocol number 2 from February 9th, 2018.

**Supplementary Information:**

The online version contains supplementary material available at 10.1186/s12888-024-06370-2.

## Background

Borderline Personality Disorder (BPD) is one of the most frequent and severe psychiatric disorders, with a prevalence of 2–3% in the general population. Direct costs related with BPD involve emergency service use, use of inpatient and outpatient treatments, prolonged sick leaves, costs related to intra-familiar abuse and neglect and in some cases legal costs [1, 2].

Meta-analyses suggest that psychological treatments are first line for problems related to BPD [3–5]. Different types of psychotherapies have shown to be effective in reducing BPD symptoms (i.e., self-harming behavior, suicidal thoughts, impulsivity, interpersonal problems, affective and cognitive symptoms). While this knowledge should benefit those affected, the reality is that dissemination of evidence-based psychotherapies in general psychiatric care is limited [6]. Barriers to dissemination include the length of training in these specialized psychotherapies, the complexity in building up a program according to such principles [7], as well as stigma attached to the population to be treated. These barriers result in an unsatisfactory health service provision for BPD: in the United States, similar to other countries, the ratio of treatment-seeking patients with this diagnosis to the professionals in evidence-based psychotherapy is insufficient (5,933:1 [6]).

In order to address this problem, two solutions are discussed: (a) developing and implementing a sufficiently effective guideline-based, treatment which is easy to learn, easy to implement and to maintain over time, such as good or general psychiatric management (GPM); (b) understanding mechanisms of change from a patient perspective, which contributes to the development of intermediate treatment goals for individual patients and helps focus on essential components of the treatment (and let go of inert elements). One of the core aspects of BPD is that the patient’s volatile emotional experience can be understood within the context of the interpersonal coherence model [7, 8]. This model assumes that the patient with BPD tends to present with idealizing and devaluating interpersonal states; these should be understood and addressed in therapy. In this case, the emerging coherence of the explanation balances out the patient’s tendency to reactive anger and impulsiveness as response to a threat to idealized relationships. The person’s sense of self – as either worthy when connected to the idealized other or worthless when threatened or rejected – directly relates with emotional processing which makes the latter a potential mechanism of change and relevant target in any treatment of BPD. In therapy, fluctuating emotional responses to interpersonal triggers (i.e., rejection, criticism, ostracism) are to be discussed in-depth by the therapist with the patient in order to raise awareness, let emerge a coherent explanation and develop alternative coping strategies. The present study assesses the effects of a brief version of good psychiatric management (GPM [8]) and investigates patient’s emotional processing as potential mechanism of change [9–11], in a controlled performance context in which the patient is put under some emotional pressure.

In the context of BPD, GPM showed promising results as comparator of Dialectical-Behavior Therapy (DBT; [12, 13]), with similar effects on all outcome indexes after one year of treatment and at 12 months follow-up. Using the personalized advantage index on a subsample, Keefe et al. [14] showed that patients with impulsive behaviors and more general symptoms benefit more from GPM, whereas patients with dependency traits and a traumatic history benefit from DBT. Brief treatment (4 months) based on GPM principles yielded promising results in terms of symptom reduction (general, borderline and interpersonal problems; [15, 16]). However, not all patients benefit from such brief GPM: a machine-learning analysis concluded that patients with high symptom load, younger age and fewer social problems benefitted the most from a 4-month GPM [17]; the patients with lower symptom load, who are older and who have more social problems benefitted less from brief GPM and may need long-term treatment.

So far, the patient-related mechanisms of change explaining outcome in GPM – as in other treatments for BPD – remain elusive, although some initial studies exist [18, 19]. Based on these studies, a review proposes six evidence-based mechanisms of change relevant for patients with BPD across treatment modalities: a) developing emotional balance (see [9–11], b) achieving interpersonal effectiveness, c) developing a stable identity, d) foster self-reflectivity, e) achieving a reality-based coherent narrative, and f) using the therapeutic relationship productively [20]. Related to a), *emotional processing* may be defined as the process by which emotions unfold and change over time from the least to the most productive, the latter being underpinned by increased meaning making and differentiation [21], thus explaining interpersonal coherence and balance. More flexible and productive emotional experiences, in response to interpersonal triggers, explain symptom decrease across psychological disorders [22].

In session analyses using observational coding of emotion carried out on GPM showed that, patients with BPD presented with lessened non-specific global distress over the course of treatment [23]. No changes in other emotion types were significant. This reduction in global distress observed in-session did not explain the outcome of treatment. In-session, the therapist-patient dyad discussed relevant interpersonal issues together – and regulated some issues related with the interpersonal coherence –, but may have foreclosed the deeper activation and elaboration of emotional experiences in the actual therapy sessions. This study assessed the in-session processes, but not the out-of-session mechanisms that could explain the outcomes and lasting changes. Doss [24] defines mechanisms of change as the interplay between the processes observed in therapy with the patient’s generalized skill level *outside of the therapy hour*, which is the variable that mediates clinical change. In this context, an independent – “therapist-free” – out-of-therapy observation context in a controlled experimental setting is needed to demonstrate a mediational path.

Activated self-criticism provides a relevant construct for the study of emotional processing outside of the therapy session [25, 26]. Patients with BPD tend to judge themselves, criticize their appearance and performance. This experience was related with self-contemptuous and self-destructive feelings [8, 27, 28], representing a vulnerability context for severe symptoms of self-harm and suicide [29, 30] in interpersonally stressful situations. Assessing emotional responses to the patient’s self-criticism outside of the therapy session – under some emotional pressure that is consistent with daily lives – is a promising avenue to observe a central mechanism of change in psychotherapy for BPD.

Taken together, these elements suggest that a brief version of good psychiatric management (GPM) may produce symptom reduction in BPD. We hypothesize that emotional processing under some pressure, assessed repeatedly in therapy-extraneous sessions focused on the patient’s self-criticism, is a mechanism of change of brief GPM. The current study was designed to test these hypotheses.

### Aims of the present study

*1. (Outcome)* A four-month BPD-specific psychiatric treatment produces more reduction in borderline symptoms than a non-specific treatment as usual (TAU). *2. (Global change)* A four-month BPD-specific psychiatric treatment presents pre-post change in emotional processing (EP) assessed under pressure, which is not the case in the TAU. *3*. *(Mediation)* The change in emotional processing (assessed between intake and mid-treatment) functions as mediator of symptom decrease (assessed between mid-treatment and discharge).

## Methods

The present randomized controlled trial (RCT) was conducted between January 2019 and October 2023 at a French-speaking outpatient setting at University Department of Psychiatry, a public mental health clinic. It is a two-arm RCT, involving a brief version of a guideline-based psychiatric treatment (GPM [8]), for BPD, over the course of a 4-month treatment program, in comparison with a 4-month treatment as usual (TAU). The study focuses on the demonstration of emotional processing as patient-related mechanism of change, assessed in an experimentally controlled context. The competent Institutional Ethics Committee, approved of the study (2017–02167). The study was registered (NCT0317818) and the protocol published [31]. In accordance with the protocol, the main hypothesis of the trial was the demonstration of the mechanism of change. Power analysis and data analytic strategy were set to test this primary hypothesis (consistent with goals 2 and 3 above), and goal (1) served as preparatory hypothesis.

Patients with Borderline Personality Disorder (BPD) according to Diagnostic and Statistical Manual of Mental Disorders, Fifth Edition [32] mastering French at a sufficient level have been included. Patients with neurocognitive disorder, psychotic spectrum disorders and bipolar disorder I were excluded from the study. In order to be able to generalize the results to a variety of clinical settings, no other exclusion criteria were applied. Diagnoses were established using SCID-5-PD [32] and were conducted by two independent raters for 25% of the cases. A mean inter-rater reliability of kappa = 0.88 for the BPD diagnoses was found.

### Patients, therapists and assessors

A total of *N* = 76 patients with BPD were included in the study among 110 patients who fulfilled study criteria (and who were screened). A total of *n* = 34 have refused to take part in the study for either unknown reasons (*n* = 21), or because of refusal of audio- and video-recording (*n* = 13). Patients agreed that data be used, should they drop out of treatment or of the research procedure. The research assessments continued according to protocol, even if the patient dropped out of treatment. According to the per-protocol power analysis with a presumed power 0.819 (for 1 mechanism, *d* = 0.60; two-tailed alpha = 0.05; 30% drop-out), this study was adequately powered for the detection of one mechanism of change (change in emotional processing). A deviation from the published protocol in terms of power and total number of *N* was required to focus on one specific mechanism instead of two (change in emotional processing and socio-cognitive processing).

A total of *N* = 28 therapists participated in the trial. They were from the same treatment context as above, and were psychiatrists (*n* = 23) and psychologists (*n* = 5) with a mean of three years of clinical experience in psychiatry. The range was between 1 and 20 years of experience. They all received at least one day of intensive workshop in GPM, as well as online material offered to learning of GPM principles. All therapists received weekly supervision by certified GPM trainers to ensure the quality control of GPM. Therapists were nested within the conditions, that means that all therapists intervened in both treatment conditions, GPM and TAU and received explicit instruction for both.

A total of *N* = 4 assessors participated in the trial. They were psychologists at the MPs, PhD or post-doc levels. They received comprehensive training in psychotherapy research methodology, the detailed assessment procedures (clinical assessment using Zanarini Rating Scale for BPD by the developer of the scale; the two-chair dialogue protocol by a certified supervisor in emotion-focused therapy), as well as in the assessments of the psychopathology and emotional processing. A program-independent researcher proceeded with the preparation of the randomization in a concealed fashion. All assessors were blind to each patient’s condition. They were polled throughout and at the end of the trial with regard to the knowledge about each patient’s condition. Correct hits were close to chance (on average 51%; range between 46% and 55%; 50% indicating chance in a two-arm RCT) and indicated that eventual knowledge of the assessors of the patient’s condition was random and non-reliable, enabling to conclude that blinding was successful.

### Interventions

Brief psychiatric treatment followed the principles of GPM [8, 34] as a straight-forward, clinically meaningful and low-resource intervention specific to BPD which may be differentiated from high-resource evidence-based psychotherapy. It encompasses the communication about the BPD diagnosis and co-morbidities, the elaboration of links between problem areas (e.g., dependency, self-harming behavior and anger), interpersonal coherence model and emotion focus, the work on treatment goals and change, and a discussion of treatment-interfering behaviors. In keeping with the GPM protocol, psychotropic medication was allowed, according to national guidelines [35]. While an example of a specific structure of brief GPM over time is provided [34], GPM is a principle-driven treatment that can adapt to a variety of clinical situations and contexts. Six principles are implemented at every session with the patient that were (a) therapist activity and structuring, (b) therapist support, (c) focus on life situations, work and relationship outside of the session, (d) therapist relationship interventions, (e) explicit expectation of change, and (f) therapist fostering patient’s accountability. Supervision was given by GPM supervisors. The last session (after four months) involved the synthesis of the main achievements and a decision for next steps in the treatment program. Patients who required more treatment were referred to either (a) additional GPM, or (b) evidence-based psychotherapy programs. The decision about the next step of treatment, if indicated, was made at this last session by the therapist-patient dyad, on the grounds of (a) the current level of symptomatology, (b) the patient’s expressed motivation, and (c) the process of change that has been observed over the four months of brief GPM.

For the TAU, non-specific crisis management as usual, safety management, and patient contact, in accordance with the minimal ethical standards and following the clinic-internal guidelines of good medical practice, was proposed. Supervision was given and supervisors received detailed instructions consistent with these internal guidelines of good medical practice. Psychotropic medication was allowed. Discussion about the BPD diagnosis, the links between problem areas and the patient’s interpersonal hypersensitivity and emotion focus, and the discussion of any interpersonal factors within BPD, were prohibited. All treatments, GPM and TAU, were delivered once-weekly, individually, in the ordinary treatment context of the public mental health outpatient clinic where the recruitment took place.

Therapist adherence to GPM principles was assessed using the self-assessment by therapist using Gunderson’s (2016; personal communication) questionnaire for adherence to GPM. This questionnaire was given to therapists in both conditions, in the end of each treatment; therapists filled them out once for each treatment. The instruction was given (in both conditions) to the therapist to adopt a self-reflective stance and to consider what was done globally over the entire four months of treatment.

### Assessments

#### Primary outcome

The primary outcome of the present study is the Zanarini Rating Scale for Borderline Personality Disorder (ZAN-BPD [33]). ZAN-BPD is a continuous hetero-administered measure assessing the nine criteria outlined in DSM-5, on a continuous Likert-type scale ranging from 0 to 4 (total score of 36). Four sub-scales have been used and are analysed: affective, cognitive, impulsivity and relationship symptoms. A validation study has shown its reliability, validity and sensitivity to change [33]. For the current study, a total of 30% of the interviews were analyzed by two trained raters, yielding an average inter-rater reliability of kappa = 0.85.

#### Secondary outcomes

Other assessments relate to intake features of the patients and secondary outcomes. They include the *Outcome Questionnaire-45* [36] which is a self-report questionnaire of 45 items aiming at assessing treatment results. It includes a global score and three sub-scales: symptomatic level, interpersonal relationship and social role. The OQ-45 has been validated in French [37]; Cronbach’s alpha for the present sample was 0.89. The *Borderline Symptom List* (BSL-23 [38], short version, is a self-report questionnaire assessing the borderline symptomatology using 23 items with satisfactory psychometric properties. The French version has shown good validity coefficients [39]. Cronbach’s alpha for the present sample was 0.94. The *Inventory of Interpersonal Problems* (IIP [40]) is a self-report questionnaire using 64 items to assess interpersonal patterns. Validity coefficients have been reported [40]. Cronbach’s alpha for the present sample was 0.90. The *Spielberger State-Trait Anger Inventory* (STAXI-2 [41]) is a self-report questionnaire on trait and state anger, using 44 items. The French validation and adaptation yielded excellent psychometric properties [42]. Cronbach’s alpha for the present sample was 0.84. The *Barratt Impulsiveness Scale* (BIS [43]) is a self-report questionnaire on the intensity of impulsivity, using 30 items. The French validation shows excellent psychometric properties [44]. Cronbach’s alpha for the present sample was 0.87. The *Difficulties in Emotion Regulation Scale* (DERS [45]) is a self-report questionnaire assessing emotion regulation using 36 items. The French translation and validation of this instrument yielded satisfactory factor structure on a student sample [46]. Cronbach’s alpha for the present sample was 0.85.

### Assessment of emotional processing

Emotional processing related to self-criticism is assessed using the experiential two-chair task focusing on the elaboration of self-criticism (as used in experiential, emotion-focused and Gestalt therapy [47]). This experiential task involves two steps (see Kramer et al., 2016; Kramer et al., 2020; Whelton et al., 2005) for the detailed description of the procedure) : (1) Imagination of a lived experience of failure and (2) Conduct of a two-chair dialogue on self-criticism related with this experience of failure, which represents an individualized and emotion-evoking procedure (Kramer et al., 2016; Whelton et al., 2005). This dialogue was video-taped and served as basis for the observer rating of the patient’s emotional processing using the Classification of Affective Meaning States (CAMS [48]). This assessment took place for all patients at intake and at 2 months into the treatment (for the current study, only these two datapoints are used), by an independent researcher.

The *Classification of Affective Meaning States* (CAMS [48]) is an observer-rated scale assessing nine distinct emotion types which can be ordered in terms of degree of emotion transformation (i.e., on the Degree of Transformation Scale, DTS [21]). The frequency of in-session emotion categories coded is based on five criteria reflecting the patient’s actual in-session performance (taking into account objective non-verbal and verbal manifestations [48]). These criteria are according to a manual [48] with specific instructions for raters to code global distress (DTS 0), fear or shame (DTS 1), rejecting anger (DTS 2), negative evaluation (DTS 3), existential need (DTS 4), assertive anger (DTS 5), self-compassion (DTS 6), hurt or grief (DTS 7), as well as acceptance and agency (DTS 8). More transformed emotional states are close to the primary adaptive emotions which indicates an increase in emotion flexibility (i.e., more diverse emotion categories in response to the self-criticism) over time, an indicator of change in emotional processing. The present study operationalizes increased emotion flexibility using the DTS [21]. The sequence assumed in the sequential model of emotional processing is plotted on an ordinal scale ranging from DTS 1 (global distress) to DTS 9 (agency and acceptance of the emotion). The current study analyzes a total of *N* = 2700 min using the CAMS. A total of 75% of the two-chair dialogues are analyzed by two trained raters, yielding an average inter-rater reliability of Intra-Class Coefficient (1, 2) = 0.78.

### Procedures

Recruitment took place at a public mental health outpatient clinic where any patient with a presumed BPD diagnosis was addressed. All screened patients met with a researcher who explained the study to them. All included patients signed the informed consent and were randomized. An independent researcher used a system involving computer-generated random numbers (block-randomization in blocks of 10) at the outset and put into sealed envelopes. Assessments took place at intake, 2 months, and discharge (follow-up was not included in the current study). According to the Data Management Plan, data was entered into RedCap on a secured space on the University server. This program allows full accountability of data management. The same Data Management Plan outlines procedures in case of adverse events (and serious adverse event) in the context of the trial which includes provision, if needed of post-trial care in case of harm. No change in the protocol was needed after the start of the trial.

Coding of the CAMS was done by additional two Master’s level students in Clinical Psychology, as well as one PhD level psychologist. They all received the equivalence of a two-day training and calibration class according to standards. They only started rating once their reliability was fully established.

### Statistical analyses

Outcomes were defined as symptom levels at discharge (or at month 2), by controlling for the relevant intake symptom level (hypothesis 1). At first, repeated measure ANOVA using Hierarchical Linear Modeling (HLM) were conducted considering the therapist random intercept in the model (thus controlling for the nesting of the patients within therapists within conditions). Then, as the variance of therapist effect was estimated zero, ANCOVAs were conducted for each of the outcomes (controlling for symptom level at intake). Statistical analyses on primary outcome were carried out both on complete cases and on Intent to Treat (including all randomized individuals) samples. For the latter, Multiple Imputation by Chained Equations (MICE) technique [49] was used to complete the missing measures by taking advantage of existing covariates such as sex. age, medication, and other comorbidities in the dataset. The result of same ANCOVA test was then pooled for 100 multiply imputed datasets using Rubin’s rule. For secondary outcomes, we decided to report only the complete cases. For hypothesis 2 (global change in emotional processing), we conducted ANOVA on the residual gains of DTS between intake and 2 months into treatment. For hypothesis 3 (mediation main hypothesis), we conducted a mediation analysis using Structural Equation Modeling for change in emotional processing as potential mechanism of change of treatment effects found under (1) between months 2 and 4. Following equations were used to construct the SEM:

$$\:\varDelta\:\:ZA{N}_{4month-2month}\sim\:b*CAM{S}_{2month-intake}+c*GPM\:\:\:\:\:\:\:\:\:\left(1\right)$$

$$\:CAM{S}_{2month-intake}\sim\:a*GPM\:\:\:\:\:\:\:\:\:\left(2\right)$$

The direct effect was defined as the estimation of coefficient *c*, and the indirect effect as multiplication of *a* and *b*. Finally, the total effect is the sum of direct and indirect effect [50](Ballen et al., 2021). To overcome the potential bias in testing the significance of these coefficients Bias-Corrected and Accelerated (BCa) Percentile Confidence Interval were calculated using 5000 bootstrap repetitions.

SPSS29 [51] and R software environment for statistical computing (Version 4.1.0) were used for all the analyses. Package “mice” and “lavaan” from R software were used for multiple imputation and mediation analysis respectively. Statistical significance level was set at *p* ≤ .05.

## Results

### Intake features of the sample

Out of the total *N* of 76 patients, *n* = 50 identified as females (for GPM: *n* = 25; for TAU: *n* = 25), and *n* = 26 as males (for GPM: *n* = 10; for TAU: *n* = 16): the distribution of sex was not different between the two conditions (Chi-Square (1) = 0.92, *p* = .47 (only binary genders were represented). The sample was composed by 95% white Caucasians, 5% other. Current co-morbid psychiatric diagnoses according to DSM-5 were not different between the two conditions (see Tables 1 and 2 for the descriptives of the sample). At intake, no primary clinical variable was different between the two conditions, with the exception of the ZAN-BPD affective sub-score with a moderate between-condition effect size at intake (*d* = 0.64; for both completer and ITT analyses; see Table 1). At the level of the secondary clinical variables, only the BIS differed between the two conditions, with a moderate between condition effect size at intake (*d* = 0.75, see Table 1). No adverse nor serious adverse effects were reported for this RCT.

**Table 1:** Descriptives of the randomized sample of patients with borderline personality disorder, per condition, at baseline (*N* = 76)

Variables	GPM (*n* = 35) M (SD)	TAU (*n* = 41) M (SD)	t	df	*p*	d
Age (years) OQ – total OQ – symptom distress OQ – interpersonal OQ- social role BSL IIP BDI STAXI BIS DERS ZAN - total ZAN – affective ZAN – cognitive ZAN – impulsivity ZAN - relationship	32.00 (10.02) 82.38 (21.72) 52.54 (15.54) 17.35 (5.56) 11.20 (5.69) 2.17 (1.07) 1.66 (0.60) 30.17 (17.45) 128.04 (19.21) 68.91 (9.99) 115.81 (25.87) 16.09 (7.02) 7.47 (2.90) 3.38 (2.35) 2.35 (2.06) 2.88 (2.01)	33.97 (10.59) 84.06 (21.03) 50.62 (13.76) 18.14 (5.01) 13.88 (5.76) 1.67 (0.80) 1.66 (0.57) 27.87 (12.81) 128.32 (15.08) 75.00 (5.84) 117.20 (34.28) 13.00 (6.22) 5.67 (2.73) 2.72 (1.98) 1.72 (1.54) 2.89 (2.00)	0.58 0.30 0.50 0.58 1.76 1.96 0.02 0.56 0.06 2.60 0.15 1.95 2.68 1.27 1.46 0.01	74 58 58 58 56 57 57 51 45 46 44 68 68 68 68 68	0.57 0.77 0.61 0.56 0.08 0.06 0.98 0.58 0.96 0.02 0.88 0.06 0.01 0.21 0.15 0.99	0.19 0.08 0.13 0.15 0.47 0.42 0.00 0.15 0.01 0.75 0.05 0.46 0.64 0.33 0.33 0.05

Note. *OQ *Outcome Questionnaire, *B* *SL *Borderline Symptom List – 23, *IIP *Inventory of Interpersonal Problems, *BDI *Beck Depression Inventory, *BIS *Barratt Impulsivity Scale, *STAXI *Spielberger Trait Anger Inventory, *DERS *Emotion Regulation Scale, *ZAN *Zanarini Scale for Borderline Personality Disorder. All measurements are at intake in this table

**Table 2:** Comorbidities of the randomized sample of patients with borderline personality disorder, per condition (*N* = 76)

Frequency of	GPM (*n* = 35) *N* (%)	TAU (*n* = 41) *N* (%)	Chi-Square	*p*
Depressive disorders Panic disorder Any anxiety disorder Substance use disorder Any eating disorder Any personality disorder	23 (66) 2 (9) 1 (3) 1 (3) 10 (29) 4 (11)	26 (63) 3 (7) 2 (5) 3 (7) 19 (46) 7 (17)	0.04 0.08 0.20 0.60 7.25 0.49	0.84 0.78 0.65 0.57 0.12 0.49

Note. All diagnoses are meant as co-morbidities in addition to diagnosed Borderline Personality Disorder. Diagnoses according to DSM-5. All degrees of freedom of 74

### Treatment integrity

Adherence to GPM was assessed in both conditions. The therapists self-reported in the GPM condition an average score to of adherence to GPM principles of 73.21 points (SD = 12.79). The therapists self-reported for the TAU condition an average adherence to GPM principles of 53.77 (SD = 11.51). This difference is statistically significant (*t* = 4.15, *p* = .001, *d* = 1.60). Medication was given in a total of *N* = 33 cases (for GPM: *n* = 13, for TAU: *n* = 20): this was not different between the conditions (Chi-Square (1) = 1.04, *p* = .36).

### Treatment attrition

Treatment attrition was defined as unilateral decision by the patient to discontinue coming to see the psychotherapist at any given time before the end of the four months of contracted treatment. For the GPM condition, *n* = 7 patients out of 35 (20%) dropped out of treatment over four months. For the TAU condition, *n* = 20 out of 41 (49%) dropped out of treatment over four months. This difference is statistically significant (Pearson’s Chi-Square (1) = 6.82, *p* = .01). The primary outcome assessments presented with an average of 16% of missing values (7% at the predefined endpoint at four months), the assessments of the mediator with an average of 24% of missing values (out of three assessment points nested within 76 patients). Importantly, patients were assessed according to protocol by the researchers despite their drop-out status.

### Pre-post changes on the ZAN-BPD

**Fig. 1:**
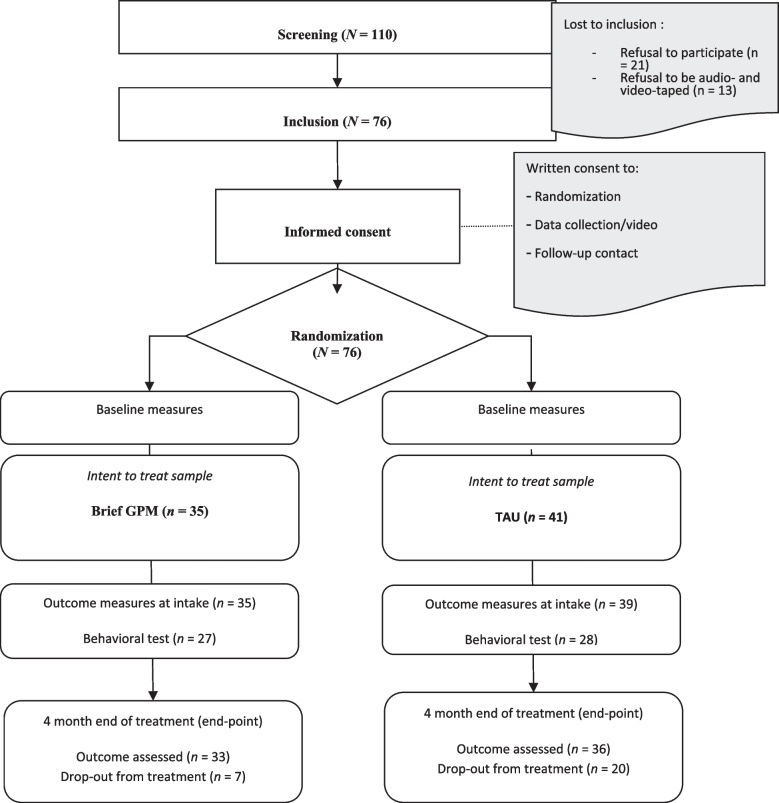
CONSORT flowchart of patient flow

For the completer analyses on the ZAN-BPD (primary outcome), we observe an overall change on average (at 2 months: ZAN-total: *t*(1, 50) = 2.29, *p* = .01, *d* = 0.33; ZAN-affective: *t*(1, 50) = 1.42, *p* = .08, *d* = 0.23; ZAN-cognitive: *t*(1, 50) = 0.62, *p* = .27, *d* = 0.08; ZAN-impulsivity; *t*(1, 50) = 1.44, *p* = .08, *d* = 0.21; ZAN-relationship; *t*(1, 50) = 3.28, *p* = .001, *d* = 0.45; at the predefined endpoint of 4 months: ZAN-total: *t*(1, 63) = 4.11, *p* = .001, *d* = 0.54; ZAN-affective: *t*(1, 63) = 3.69, *p* = .001, *d* = 0.50; ZAN-cognitive: *t*(1, 63) = 3.35, *p* = .001, *d* = 0.42; ZAN-impulsivity; *t*(1, 63) = 1.86, *p* = .03, *d* = 0.31; ZAN-relationship; *t*(1, 63) = 2.72, *p* = .01, *d* = 0.39) (Fig. 1).

### Effects of GPM on the ZAN-BPD

**Fig. 2:**
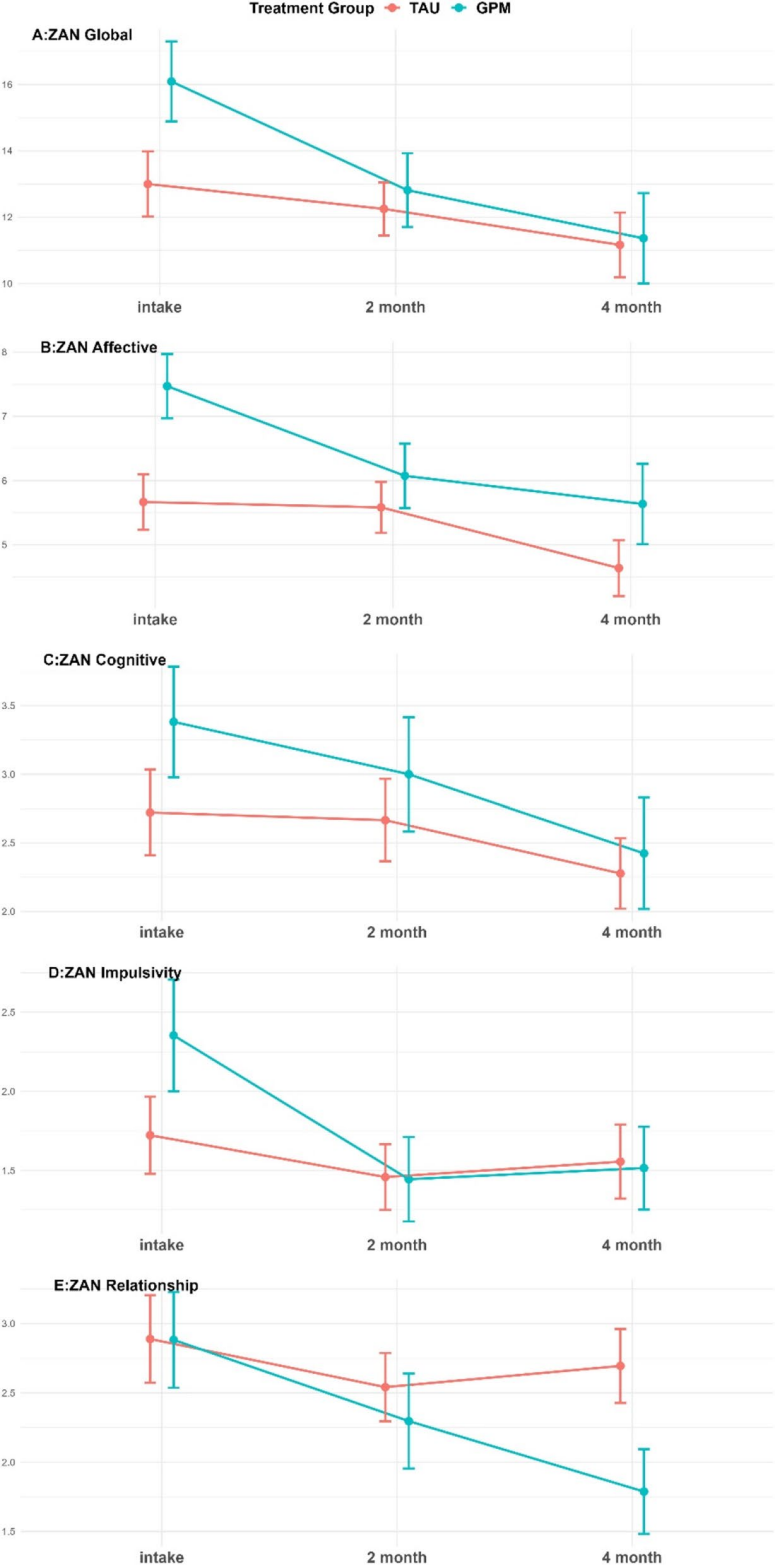
Borderline symptom change over four months of treatment in borderline symptoms comparing GPM with TAU. *Note.* Effect size *d* is computed as between-condition effect at the end-point (after 4 months).

As regards the main hypothesis of effect of GPM, with multiple imputations to account for missing values in the main outcomes, in comparison with TAU, no between condition-difference was found for the ZAN-BPD total score (at 2 months: ZAN-total: *F*(1, 49) = 0.30, *p* = .59, *d* = 0.25; ZAN-affective: *t*(1, 49) = 0.36, *p* = .55, *d* = 0.38; ZAN-cognitive: *t*(1, 49) = 0.43, *p* = .51, *d* = 0.20; ZAN-impulsivity; *t*(1, 49) = 0.22, *p* = .64, *d* = 0.12; ZAN-relationship; *t*(1, 49) = 0.29, *p* = .59, *d* = 0.05; at the predefined endpoint of 4 months: ZAN-total: *t*(1, 63) = 0.08, *p* = .78, *d* = 0.04; ZAN-affective: *t*(1, 63) = 1.54, *p* = .21, *d* = 0.17; ZAN-cognitive: *t*(1, 63) = 0.11, *p* = .74, *d* = 0.11; ZAN-impulsivity; *t*(1, 63) = 0.05, *p* = .82, *d* = 0.01; ZAN-relationship; *t*(1, 63) = 4.48, *p* = .03, *d* = 0.56; see Fig. 2). Consistent patterns of results were found with and without multiply imputed data and the pooled tests (see Tables 3 and 4 for the non-imputed results). All models were adjusted for the respective score at intake.

**Table 3:** Symptom levels in brief psychiatric management for borderline personality disorder, intent-to-treat analyses after two months of treatment (*N* = 74; intent-to-Treat)

Variables	GPM (*n* = 35) M (SD)	TAU (*n* = 39) M (SD)	F	*p*	d
ZAN - total ZAN – affective ZAN – cognitive ZAN – impulsivity ZAN - relationship OQ – total OQ – symptom distress OQ – interpersonal OQ- social role BSL IIP BDI STAXI BIS DERS	13.83 (7.45) 6.54 (3.21) 3.14 (2.48) 1.86 (2.00) 2.29 (1.95) 84.32 (28.66) 51.57 (18.44) 18.46 (6.74) 13.22 (6.41) 1.90 (1.16) 1.60 (0.70) 29.18 (19.45) 124.33 (23.16) 67.22 (11.07) 108.81 (28.00)	12.15 (6.00) 5.44 (2.62) 2.69 (1.95) 1.64 (1.61) 2.38 (1.71) 84.97 (20.73) 50.80 (13.94) 18.49 (4.85) 13.42 (5.90) 1.67 (0.80) 1.69 (0.50) 27.81 (12.67) 128.52 (15.87) 77.44 (11.01) 119.72 (35.66)	0.11 0.51 0.39 0.00 0.83 0.12 0.09 0.38 0.11 0.56 2.30 0.85 0.94 5.44 0.22	0.75 0.48 0.54 0.99 0.37 0.74 0.77 0.54 0.74 0.46 0.14 0.37 0.34 0.03 0.64	0.25 0.38 0.20 0.12 0.05 0.03 0.05 0.00 0.03 0.23 0.15 0.08 0.21 0.93 0.34

Note. All analyses control for the same measure at intake. ANCOVA was used to test the effect of treatment. Two patients did not have data at intake for symptom assessments

**Table 4:** Symptom levels in brief psychiatric management for borderline personality disorder, intent-to-treat analyses after four months of treatment (*N* = 74; intent-to-Treat)

Variables	GPM (*n* = 35) M (SD)	TAU (*n* = 39) M (SD)	F	*p*	d
ZAN - total ZAN – affective ZAN – cognitive ZAN – impulsivity ZAN - relationship OQ – total OQ – symptom distress OQ – interpersonal OQ- social role BSL IIP BDI STAXI BIS DERS	11.34 (7.70) 5.66 (2.64) 2.49 (2.33) 1.49 (1.50) 1.71 (1.76) 78.92 (31.53) 46.82 (21.17) 16.86 (7.06) 13.54 (5.20) 1.73 (1.21) 1.48 (0.70) 28.50 (18.92) 121.11 (22.72) 68.91 (9.99) 94.50 (34.96)	11.03 (6.02) 4.68 (7.70) 2.28 (1.61) 1.48 (1.45) 2.65 (1.63) 85.60 (19.41) 50.37 (13.50) 18.51 (4.65) 14.49 (5.20) 1.69 (0.72) 1.64 (0.52) 27.55 (12.34) 127.23 (16.94) 75.00 (5.84) 104.00 (35.81)	0.02 1.08 0.02 0.06 5.53 1.12 0.33 1.63 4.42 0.56 2.71 0.00 3.60 1.59 0.52	0.89 0.30 0.89 0.81 0.02 0.30 0.58 0.22 0.04 0.46 0.11 0.97 0.07 0.22 0.48	0.04 0.17 0.11 0.01 0.56 0.26 0.20 0.28 0.49 0.04 0.26 0.06 0.31 0.74 0.32

Note. All analyses control for the same measure at intake. ANCOVA used for the four sub-scales of ZAN-BPD. Two patients did not have data at intake for symptom assessments

### Secondary outcomes

For the secondary outcomes (only the more conservative intent to treat analyses are reported), Tables 3 and 4 report analyses for all secondary outcomes and found significant and small, between condition effects for STAXI at two months (*d* = 0.21), and for STAXI (*d* = 0.31), OQ-total (*d* = 0.20), OQ-symptom distress (*d* = 0.28) and for IIP (*d* = 0.26) at four months, all favoring GPM, in comparison with TAU. Moderate to large between-condition effects were systematically found for BIS for all time points, despite controlling for intake variables (0.74 < *d* < 0.93), favoring GPM.

### Change in emotional processing assessed in a series of two-chair dialogues over time

The second hypothesis concerned the global change on emotional processing assessed in an experiential two-chair dialogue, between intake and 2 months. Of note, due to missing data at the level of the assessed mechanism, the total *n* for this analysis is 50 (*n* = 26 patients with missing data point(s) at either month 2 or month 4; we excluded them listwise from this analysis). The average DTS of emotional processing for intake (GPM: 0.63 (SD = 1.06); TAU: 0.92 (SD = 1.39)) and 2 months (GPM: 4.63 (SD = 2.08); TAU: 3.30 (SD = 2.23)). Condition had a medium to large effect on the DTS of emotional processing assessed under emotional pressure, favoring more productive change in GPM (*t*(1, 48) = 2.06, *p* = .01; *d* = 0.74).

### Mediation of emotional processing to explain symptom change

**Fig. 3:**
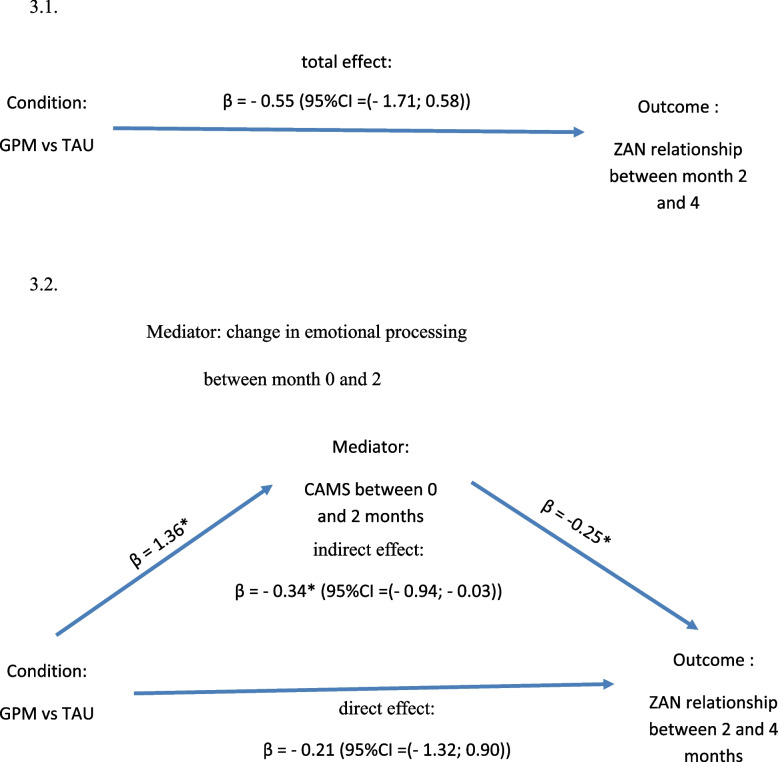
Emotional processing assessed under emotional pressure mediates the effect of brief psychiatric treatment

The third and main hypothesis focused on the mediating effect. A regression-based procedure using SEM was carried out as illustrated in Fig. 3. Consistent with the findings presented above, the indirect effect of condition on the reduction in ZAN-BPD subscale relationship problems between 2 months and 4 months (discharge) was significantly mediated by the change in DTS of emotional processing between intake and 2 months. The 95% confidence interval for indirect effect did not contain the null value (β = −0.34; 95%CI = (− 0.94; − 0.03)). However, the indirect effect of condition on the reduction in ZAN-BPD subscale relationship problems between 2 months and 4 months (discharge) was not significant (β =- 0.21; 95% CI = (− 1.32; 0.90)). The Total effect was calculated as the sum of two previous effects (β = − 0.55 ; 95% CI = (− 1.71; 0.58)).

## Discussion

The goal of the present study was to determine the mechanism of change in brief psychiatric intervention according to GPM. In order to address this main goal, we aimed to demonstrate, firstly, the effectiveness of a brief version of Good Psychiatric Management (GPM over four months) for BPD, as compared to non-specific treatment as usual on the intensity of BPD symptoms. Secondly, we aimed at demonstrating that emotional processing assessed in the context of a controlled out-of-session paradigm eliciting self-criticism in the patient (under some emotional pressure, consistent with the interpersonal coherence model) increased over GPM. Thirdly, the main hypothesis assumed the mediation of the treatment effects by emotional processing [31]. The results were partially consistent with the hypotheses, showing no effect of GPM on the main score of ZAN-BPD, but a specific reduction of brief GPM on the ZAN-BPD relationship sub-scale and evidence for change in emotional processing as mechanism of change of these effects. Given the scarce resources and the lack of evidence-based psychotherapies for BPD across contexts [6, 7], brief GPM may be an important treatment option in particular if relationship problems need to be addressed.

On the overall score of the ZAN-BPD, there was no between-condition effect, which was also reflected in the small (non-significant) effects observed at all measurement times for the ZAN-BPD sub-scales affective, cognitive and impulsivity. We assume that the present study laid the bar for between-condition effect high with a nested design in which all therapists were involved in both treatment arms. It could be argued that there were carry-over effects from GPM to TAU, as it can be assumed that GPM may have become a default clinical intervention in all trained therapists and it may be difficult for a clinician not to apply it once trained. Therapist adherence to GPM principles in the GPM condition was satisfactory and therapist adherence to GPM principles was close to chance in the TAU, which suggests that such carry-over effects, if present at all, were small and negligible. Symptom intensity on the ZAN-BPD at intake was moderate, and somewhat lower than in other trials involving GPM (see [12]). This specificity may also have yielded an average floor effect in which patients, on top of the natural remittive course of symptoms over time, had rather small disorder-specific gains to make, which remained undetected in the current sample. The effect of GPM may be more specific and limited to areas related with the interpersonal coherence model.

On the level of the secondary outcomes, GPM over four months yielded a positive impact on treatment retention, and a medium size effect on the specific relationship difficulties in BPD, in addition to reducing social problems and impulsivity. Taken together, brief GPM can be considered as a potentially effective treatment for BPD, in particular for treating relationship problems, impulsivity and social problems, which is consistent with earlier studies [12–16]. Finding reliable symptom reduction after such a brief time (i.e., four months of treatment) is consistent with emerging literature in the domain of BPD for several brief versions of evidence-based psychotherapies, such as six-months DBT [52] and mentalization-based treatment [53]. In these two trials, effectiveness of the brief versions was comparable to the longer, standard, versions of the evidence-based psychotherapies. Additional research, for example on STEPPS [54], shows consistent results for treatments lasting six months. Compared to these more complex and high-resource therapy approaches, GPM, in particular brief formats in the context of stepped care [55, 56], may have the advantage to be more easily disseminable across contexts, but may have the disadvantage of a more limited impact, when compared to brief versions of evidence-based psychotherapies. While a formal test of this question awaits further research, we hypothesize that brief GPM may be specifically effective to treat BPD’s acute relationship problems, and more time spent in therapy may be needed to treat the full array of borderline symptoms and comorbidities [17, 57]. More such outcome studies need to be carried out, in order to respond to the critical question of dissemination of good-enough treatment principles for BPD [6].

Determining how psychotherapy works is particularly critical in the context of severe personality disorders, such as BPD [18–20, 58]. Determining evidence for mechanisms of change is crucial to develop individualized intermediate treatment targets that can be used from a variety of therapists across treatment types [59]. Consistent with GPM’s interpersonal coherence model [8], in the present study, we found that change in emotional processing related to an episode of self-criticism assessed early in treatment and outside of the session (consistent with [24]), explains the reduction of relationship problems a few weeks later. The Degree of Transformation Score (DTS) in the GPM condition (at month 2) was on the level of the individual’s access to their needs in response to their self-criticism, while the DTS in TAU remained on the expression of their negative evaluation. This difference is significant (and explains relationship change two months later), but most of all this difference is clinically significant: it is consistent with the interpersonal coherence model [8]. Helping the patient to gain clarity over their activated emotional experiences, as they happen in the moment is conducive of increased interpersonal clarity and competence, supported by the GPM therapist discussing self-other interactions and affective experiences, contributing to diminishing patient’s dependent behavior and anger. More broadly, the patient’s access to deeper emotional processing is at the core of effective therapy from a transtheoretical perspective [9–11].

The present study is one of the few which differentiated the measurement window of the mechanism of change (assessed here between intake and two months) from the measurement window of the outcome (here done right after, between two and four months into the treatment [60], enabling to separate potential explanations from their consequences. In addition, this study assessed emotional change using validated observer-rated scales and thus, the results come from theory-informed process observation of the patient’s out-of-session performance under some emotional pressure. There are two advantages of such a design: a) the internal validity is high with a theory-informed assessment context, and b) the ecological validity is presumably high, as it was observed that the patients with BPD experience moments of distress and low self-esteem in their daily lives [28, 29, 61]. The results suggest that emotions that are experienced by patients with BPD may only explain symptom change under certain conditions: when the therapist explicitly establishes links between the patient’s emotional responses and their interpersonal hypersensitivity or fear of rejection, ostracism or exclusion [8]. This strengthens the notion that the intermediate goal for a patient with BPD of reaching emotional balance within the relevant interpersonal context, may be understood as an evidence-based core mechanism of change [20].

A number of limitations need to be acknowledged. Although the sample was small, the study was sufficiently powered to demonstrate reliable effects. Given the focus on the mechanism of change, the outcome part of the present study should be replicated with a particular focus on the most promising symptom domains of relationship problems and impulsivity. The predefined outcome was ZAN-BPD without specification of the sub-scales, in the current analysis, we offer both analysis on the main score and of the sub-scales (without pre-definition). Severity of the overall personality disorder was not assessed using dimensional (or categorical) constructs. Adherence to GPM principles was a therapist self-reported measure: an observer rated approach may be needed to assess the more precise level of adherence and competence in both conditions. In particular, the use of specific GPM relationship interventions, and the use of the real relationship in GPM, may be more suitably assessed using observer-rated instruments. The assessment of emotional processing under some pressure presented a variety of intensities across patients, which was not controlled for in the present study (i.e., intensity of self-contempt, for example [27]). Follow-up analyses, including the usage of further treatment, at one year have not yet been included in the present study. Given that for the mediator, the number of missing values was high, it was remarkable to find a significant path, but we should remain cautious with its interpretation due to possible retention biases. The mediation path over emotional processing may have been moderated by (a) the patient’s intake of emotion regulation capacities and/or (b) the quality of the therapeutic alliance, which would request a moderated mediation approach; the a priori statistical power was based on a mediation model without moderator. We did not analyze a concurrent concept for mediation (e.g., socio-cognitive processing), as recommended [60].

## Conclusions

In conclusion, effective psychotherapies of BPD exist, but they remain difficult to disseminate due to their complexity. Brief psychiatric treatment may be a cost-effective complement and may represent a promising initial treatment step within a stepped care approach, specifically for patients with relationship problems: its implementation may therefore be a major priority in the mental health care system, reducing waitlists for evidence-based psychotherapies. This is the first study formally testing the effectiveness of such brief psychiatric treatment compared to a TAU *and* its underlying mechanism of change. To conclude, brief psychiatric treatment according to GPM may work through the patient’s processing of more healthy emotions when faced with self-critical events, in the context of a sufficiently efficient care that directly focuses on the interpersonal vulnerability of BPD.

## Supplementary Information

Supplementary Material 1

Supplementary Material 2

## Data Availability

The datasets used during the current study are available from the corresponding author on reasonable request.

## Abbreviations

BPD: borderline personality disorder
GPM: Good Psychiatric Management
RCT: Randomized Controlled Trial
TAU: Treatment as Usual
ZAN-BPD: Zanarini Rating Scale for Borderline Personality Disorder
OQ-45: Outcome Questionnaire-45
BSL-23: Borderline Symptom List-23
IIP: Inventory of Interpersonal Problems
STAXI: Spielberger State-Trait Anger Inventory
BIS: Barratt Impulsiveness Scale
DERS: Difficulties in Emotion Regulation
CAMS: Classification of Affective Meaning States
DTS: Degree of Transformation Scale
