# Supplementary material for: Emotional processing as mechanism of change in brief good psychiatric management for borderline personality disorder: results of a randomized controlled trial
Source: BMC Psychiatry. 2024 Dec 18;24:921. doi: 10.1186/s12888-024-06370-2 (PMC11656953; doi:10.1186/s12888-024-06370-2)
Supplement: Supplementary file 1 — Supplementary Material 1 [file 12888_2024_6370_MOESM1_ESM.docx]

SUPPLEMENTARY MATERIAL

Table 1

Descriptives of the randomized sample of patients with borderline personality disorder, per condition (*N* = 76)

| Variables | GPM (*n* = 35)  M (SD) | TAU (*n* = 41)  M (SD) | *t* | *df* | *p* | *d* |
| --- | --- | --- | --- | --- | --- | --- |
| Age (years)  OQ – total  OQ – symptom distress  OQ – interpersonal  OQ- social role  BSL  IIP  BDI  STAXI  BIS  DERS  ZAN - total  ZAN – affective  ZAN – cognitive  ZAN – impulsivity  ZAN - relationship | 32.00 (10.02)  82.38 (21.72)  52.54 (15.54)  17.35 (5.56)  11.20 (5.69)  2.17 (1.07)  1.66 (0.60)  30.17 (17.45)  128.04 (19.21)  68.91 (9.99)  115.81 (25.87)  16.09 (7.02)  7.47 (2.90)  3.38 (2.35)  2.35 (2.06)  2.88 (2.01) | 33.97 (10.59)  84.06 (21.03)  50.62 (13.76)  18.14 (5.01)  13.88 (5.76)  1.67 (0.80)  1.66 (0.57)  27.87 (12.81)  128.32 (15.08)  75.00 (5.84)  117.20 (34.28)  13.00 (6.22)  5.67 (2.73)  2.72 (1.98)  1.72 (1.54)  2.89 (2.00) | 0.58  0.30  0.50  0.58  1.76  1.96  0.02  0.56  0.06  2.60  0.15  1.95  2.68  1.27  1.46  0.01 | 74  58  58  58  56  57  57  51  45  46  44  68  68  68  68  68 | .57  .77  .61  .56  .08  .06  .98  .58  .96  .02  .88  .06  .01  .21  .15  .99 | 0.19  0.08  0.13  0.15  0.47  0.42  0.00  0.15  0.01  0.75  0.05  0.46  0.64  0.33  0.33  0.05 |

*Note.* OQ: Outcome Questionnaire; BSL: Borderline Symptom List – 23 ; IIP : Inventory of Interpersonal Problems; BDI: Beck Depression Inventory; BIS: Barratt Impulsivity Scale; STAXI: Spielberger Trait Anger Inventory; DERS: Emotion Regulation Scale; ZAN: Zanarini Scale for Borderline Personality Disorder.

Table 2

Comorbidities of the randomized sample of patients with borderline personality disorder, per condition (*N* = 76)

| Frequency of | GPM (*n* = 35)  *N (%)* | TAU (*n* = 41)  *N (%)* | *Chi-Square* | *p* |
| --- | --- | --- | --- | --- |
| Depressive disorders  Panic disorder  Any anxiety disorder  Substance use disorder  Any eating disorder  Any personality disorder | 23 (66)  2 (9)  1 (3)  1 (3)  10 (29)  4 (11) | 26 (63)  3 (7)  2 (5)  3 (7)  19 (46)  7 (17) | 0.04  0.08  0.20  0.60  7.25  0.49 | .84  .78  .65  .57  .12  .49 |

*Note*. All diagnoses are meant as co-morbidities in addition to diagnosed Borderline Personality Disorder. Diagnoses according to DSM-5. All degrees of freedom of 74.

Table 3

Symptom change in brief psychiatric management for borderline personality disorder, intent-to-treat analyses after two months of treatment (*N* = 74)

| Variables | GPM (*n* = 35)  M (SD) | TAU (*n* = 39)  M (SD) | *F* | *p* | *d* |
| --- | --- | --- | --- | --- | --- |
| ZAN - total  ZAN – affective  ZAN – cognitive  ZAN – impulsivity  ZAN - relationship  OQ – total  OQ – symptom distress  OQ – interpersonal  OQ- social role  BSL  IIP  BDI  STAXI  BIS  DERS | 13.83 (7.45)  6.54 (3.21)  3.14 (2.48)  1.86 (2.00)  2.29 (1.95)  84.32 (28.66)  51.57 (18.44)  18.46 (6.74)  13.22 (6.41)  1.90 (1.16)  1.60 (0.70)  29.18 (19.45)  124.33 (23.16)  67.22 (11.07)  108.81 (28.00) | 12.15 (6.00)  5.44 (2.62)  2.69 (1.95)  1.64 (1.61)  2.38 (1.71)  84.97 (20.73)  50.80 (13.94)  18.49 (4.85)  13.42 (5.90)  1.67 (0.80)  1.69 (0.50)  27.81 (12.67)  128.52 (15.87)  77.44 (11.01)  119.72 (35.66) | 2.42  2.32  1.18  0.69  0.22  1.81  3.21  0.42  0.01  0.09  3.63  1.05  4.69  13.92  3.70 | .13  .14  .28  .41  .65  .19  .08  .52  .93  .77  .06  .31  .04  .001  .06 | 0.25  0.38  0.20  0.12  0.05  0.03  0.05  0.00  0.03  0.23  0.15  0.08  0.21  0.93  0.34 |

*Note*. All analyses control for ZAN affective at intake. MANCOVA used for the four sub-scales of ZAN-BPD. Two patients did not have data at intake for symptom assessments.
